# Carbachol-mediated pigment granule dispersion in retinal pigment epithelium requires Ca^2+ ^and calcineurin

**DOI:** 10.1186/1471-2121-8-53

**Published:** 2007-12-19

**Authors:** Adam S Johnson, Dana M García

**Affiliations:** 1 Biology Department, Texas State University-San Marcos, San Marcos, Texas 78666, USA

## Abstract

**Background:**

Inside bluegill (*Lepomis macrochirus*) retinal pigment epithelial cells, pigment granules move in response to extracellular signals. During the process of aggregation, pigment motility is directed toward the cell nucleus; in dispersion, pigment is directed away from the nucleus and into long apical processes. A number of different chemicals have been found to initiate dispersion, and carbachol (an acetylcholine analog) is one example. Previous research indicates that the carbachol-receptor interaction activates a G_q_-mediated pathway which is commonly linked to Ca^2+ ^mobilization. The purpose of the present study was to test for involvement of calcium and to probe calcium-dependent mediators to reveal their role in carbachol-mediated dispersion.

**Results:**

Carbachol-induced pigment granule dispersion was blocked by the calcium chelator BAPTA. In contrast, the calcium channel antagonist verapamil, and incubation in Ca^2+^-free medium failed to block carbachol-induced dispersion. The calcineurin inhibitor cypermethrin blocked carbachol-induced dispersion; whereas, two protein kinase C inhibitors (staurosporine and bisindolylmaleimide II) failed to block carbachol-induced dispersion, and the protein kinase C activator phorbol 12-myristate 13-acetate failed to elicit dispersion.

**Conclusion:**

A rise in intracellular calcium is necessary for carbachol-induced dispersion; however, the Ca^2+ ^requirement is not dependent on extracellular sources, implying that intracellular stores are sufficient to enable pigment granule dispersion to occur. Calcineurin is a likely Ca^2+^-dependent mediator involved in the signal cascade. Although the pathway leads to the generation of diacylglycerol and calcium (both required for the activation of certain PKC isoforms), our evidence does not support a significant role for PKC.

## Background

Organelle motility is an essential function of all cells. The shuttling of supramolecular structures is regulated by motor proteins, cytoskeletal elements, and a wide variety of chemical messengers. Pigment cells are an excellent model in which to study cell motility because pigment granules are readily visible, move rapidly, and undergo reversible movements which can be manipulated experimentally [1]. Found in a variety of cell types, pigment granule motility in the retinal pigment epithelium (RPE) was examined in the present study.

**Figure 1:**
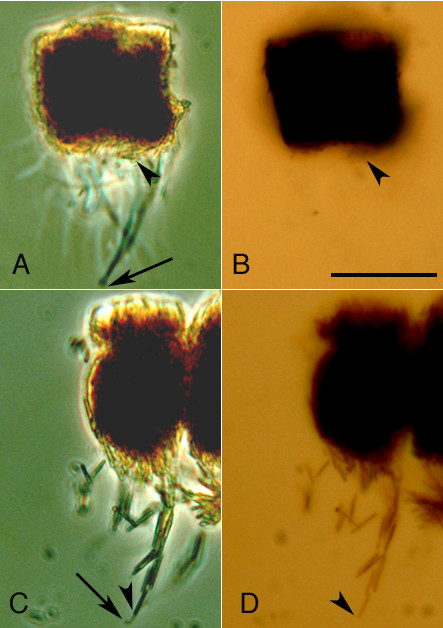
**RPE with aggregated and dispersed pigment granules**. RPE cells with aggregated pigment granules possess apical processes which appear dark grey in phase contrast micrographs (A). In brightfield micrographs, the processes empty of melanosomes are invisible (B). In contrast, RPE with dispersed pigment granules have processes filled with pigment granules which when viewed with phase contrast optics (C) are refractile and appear bright in some cases (although individual granules cannot be resolved). In brightfield micrographs, the same pigment granules appear brown. Arrowheads indicate the position of distal pigment granules while arrows point to the tips of processes (phase contrast micrographs only). The scale bar represents 20 micrometers.

The RPE is a single layer of cells found between the neural retina and the choroid. In animals that do not possess the ability to constrict the pupil, RPE cells possess apical processes which interdigitate with photoreceptors [2,3]. Within each cell, pigment granules aggregate and disperse. In the aggregated state, pigment granules are withdrawn from the apical processes and cluster in the cell body (Figure 1A–B), while in the dispersed state, they are moved down the lengths of apical processes as shown in Figure 1C–D. In the dispersed state, protection of rod photoreceptors from photobleaching is thought to be enhanced [3-6].

Extracellular molecular mediators stimulate pigment granule motility, and several different agents have been identified that induce movement. Forskolin (FSK) stimulates adenylyl cyclase to increase intracellular levels of cAMP, resulting in aggregation [7-14]. Catecholamines and their agonists (epinephrine, phenylephrine, clonidine, apomorphine, and dopamine) induce dispersion [9,13,15]. Dopamine acts through D2 receptors which inhibit adenylyl cyclase [13]. With adenylyl cyclase inhibited, [cAMP]_i _decreases and dispersion ensues.

Catecholamines are not the only extracellular messengers that induce pigment granule dispersion in RPE. In 1998, García [16] reported that the acetylcholine analog carbachol induces pigment granule dispersion in RPE isolated from green sunfish (*Lepomis cyanellus*). González *et al*. [17] extended this finding to RPE isolated from bluegill (*Lepomis macrochirus*) and further reported that muscarinic M_odd _receptor activation leads to pigment granule motility. Later it was found that the native ligand acetylcholine induces pigment granule dispersion [18]. Following M_odd _receptor activation, phospholipase C is activated, cleaving PIP_2 _to generate diacylglycerol and inositol trisphosphate (IP_3_). Antagonists to the IP_3 _receptor inhibited carbachol-induced dispersion [18].

In other systems, the IP_3 _receptor has been found within the membrane of the endoplasmic reticulum. With ligand bound to the IP_3 _receptor, Ca^2+ ^stored within the ER lumen is released into the cytosol (see [19]). Extrapolating these observations to regulation of pigment granule movement in RPE, one might infer a role for Ca^2+ ^in regulating pigment granule dispersion in RPE. However, King-Smith *et al*. [20] were unable to demonstrate a role for Ca^2+ ^in either pigment granule dispersion or aggregation. Rather, they found that when dispersion is experimentally induced by cAMP washout in RPE isolated from green sunfish (*Lepomis cyanellus*), there is no significant rise in intracellular Ca^2+ ^levels nor does chelating Ca^2+ ^prevent pigment granule dispersion. This finding may not, however, rule out a role for Ca^2+ ^in a physiological setting involving receptor activation if the physiological role for Ca^2+ ^precedes a decrease in intracellular [cAMP]. Therefore, the hypothesis driving our study was that Ca^2+ ^is required for carbachol-induced dispersion in RPE cells isolated from bluegill, which we examined using calcium chelators, calcium channel blockers and calcium-free media. Furthermore, we predicted that if calcium were required, calcium-dependent effectors would also be expected to play a role in pigment granule dispersion; therefore, intracellular mediators involved in pigment granule dispersion were explored.

## Results

### Exploration for calcium requirements in carbachol-induced pigment granule dispersion

**Figure 2:**
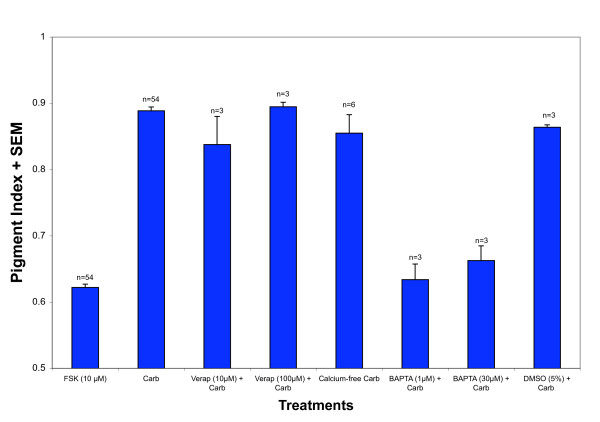
**Carbachol-induced dispersion requires intracellular, but not extracellular, Ca^2+ ^sources**. Isolated RPE cells were first treated with 10 μM forskolin for 45' to induce aggregation. Following aggregation, cells were treated with 100 nM carbachol (Carb) along with verapamil (Ver), BAPTA-AM (BAPTA) or DMSO as a carrier control for BAPTA-AM. The mean pigment index of cells treated with BAPTA-AM was significantly different than the mean pigment index of cells treated with carbachol alone. Dispersion was inhibited 95% in cells treated with 1 μM BAPTA and 82% in those treated with 30 μM BAPTA. No statistically significant differences were found between the FSK, BAPTA at 1 μM, or BAPTA at 30 μM treatments. As for verapamil-treated cells, no inhibition of dispersion was observed, nor was dispersion inhibited in DMSO controls. In addition, 100 nM carbachol was applied to cells in either Ringer or in Ca^2+^-free Ringer. The mean pigment index of the Ca^2+^-free treatment was not different statistically from the carbachol control. The error bars represent the standard error of the mean (SEM).

To test whether an elevation in intracellular Ca^2+ ^was required for carbachol-induced pigment granule dispersion, RPE cells were isolated, treated with forskolin (FSK) and then challenged to disperse in the presence or absence of the membrane-permeant Ca^2+ ^chelator BAPTA-AM. When cells were incubated with 100 nM carbachol in the presence of 1 μM BAPTA-AM or 30 μM BAPTA-AM, the resulting pigment index (PI) values (0.63 ± 0.02, n = 3; 0.66 ± 0.02, n = 3, respectively) were not statistically significantly different from the PI of cells treated with FSK (0.62 ± 0.01, n = 54) but were different from PI of cells treated with carbachol alone (PI = 0.89 ± 0.01, n = 54) (Figure 2). The maximum concentration of DMSO used during incubation (5% vol/vol) did not alter PI values in controls (PI carbachol = 0.89 ± 0.01, n = 54; PI carbachol in 5% DMSO = 0.86 ± 0.00, n = 3).

To test the importance of extracellular Ca^2+ ^in carbachol-induced pigment granule dispersion, verapamil, an L-type Ca^2+ ^channel antagonist, was tested for its ability to block carbachol-induced dispersion. Verapamil failed to block carbachol-induced dispersion at both 10 μM (PI = 0.84 ± 0.04, n = 3) and 100 μM (0.89 ± 0.01, n = 3) concentrations. The difference in PI between cells with and without the Ca^2+ ^channel blocker was not statistically significant (Figure 2). In addition, the differences between the pigment indices for either condition (carbachol or carbachol plus verapamil) and the FSK condition were statistically significant.

To confirm that an influx of Ca^2+ ^from the extracellular medium was not required, RPE cells were isolated, treated with forskolin (FSK) and then challenged to disperse in the presence or absence of extracellular Ca^2+^. RPE were treated with 10 μM FSK to induce pigment granule aggregation followed by carbachol (100 nM) to induce pigment granules to disperse. The difference in PI between cells with (0.89 ± 0.01, n = 54) and without (0.86 ± 0.03, n = 6) extracellular Ca^2+ ^available was not statistically significant (Figure 2). However, the difference between either condition (with and without Ca^2+^) and the 10 μM FSK condition was statistically significantly different.

### Investigation of calcium-dependent mediator proteins

**Figure 3:**
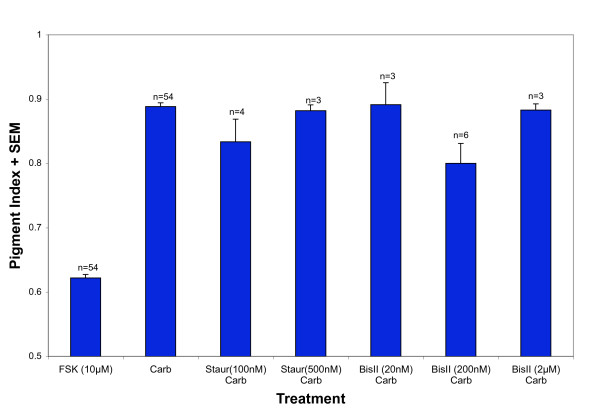
**Inhibitors of PKC do not significantly inhibit carbachol-induced dispersion**. Isolated RPE cells were first treated with 10 μM forskolin for 45' to induce aggregation. Following aggregation, cells were treated with 100 nM carbachol (Carb) along with either 100 nM or 500 nM staurosporine (Staur), a PKC-inhibitor. The mean pigment indices of cells treated with staurosporine at these concentrations were not statistically significantly different than the mean pigment index of cells treated with carbachol alone. Bisindolylmaleimide II (BisII), a second PKC-inhibitor, was also applied to cells in the presence of carbachol following aggregation. The pigment index of cells treated with 20 nM or 2 μM bisindolylmaleimide II was not significantly different from the pigment index of cells treated with carbachol alone. However, the pigment index of cells treated with 200 nM bisindolylmaleimide was statistically significantly different, and dispersion was inhibited by 20%.

Since our results using BAPTA-AM suggested a role for intracellular Ca^2+^, we tested possible downstream, Ca^2+^-dependent mediators. We first tested whether protein kinase C (PKC) is required for carbachol-induced dispersion by using two inhibitors (staurosporine and bisindolylmaleimide II). Cells treated with carbachol in the absence or presence of staurosporine (100 nM, 0.83 ± 0.04, n = 4; 500 nM, 0.88 ± 0.01, n = 3) were found to differ from the cells treated with FSK, but were not different from each other (Figure 3). When bisindolylmaleimide II (20 nM, 0.89 ± 0.03, n = 3; 200 nM, 0.80 ± 0.03, n = 6; 2 μM, 0.88 ± 0.01, n = 3) was applied with carbachol, dispersion was partially inhibited, but only in the 200 nM treatment (Figure 3). Exhibiting a 20% inhibition in dispersion, the cells treated with 200 nM bisindolylmaleimide II were statistically significantly different from both FSK and carbachol treatments.

**Figure 4:**
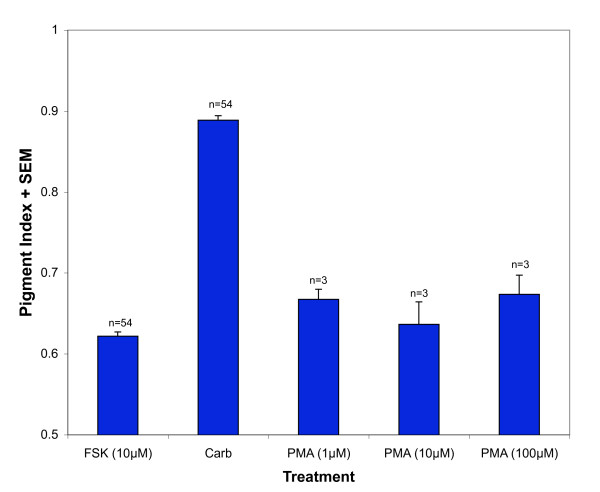
**PKC activator PMA did not induce dispersion**. Isolated RPE cells were first treated with 10 μM forskolin for 45' to induce aggregation. Following aggregation, cells were either treated with carbachol (100 nM) or with PMA, a PKC activator. The pigment indices of cells treated with PMA were significantly different from the pigment index of cells treated with carbachol and were not significantly different from the pigment index of cells treated with forskolin, indicating that PMA failed to induce pigment granule dispersion.

To test whether activating PKC was sufficient to induce dispersion, PMA was applied to RPE. Following FSK treatment, PMA (without carbachol) elicited PI values (1 μM, 0.67 ± 0.01, n = 3; 10 μM, 0.64 ± 0.03, n = 3; 100 μM, 0.67 ± 0.02, n = 3) that were not significantly different from FSK-treated cells (Figure 4).

**Figure 5:**
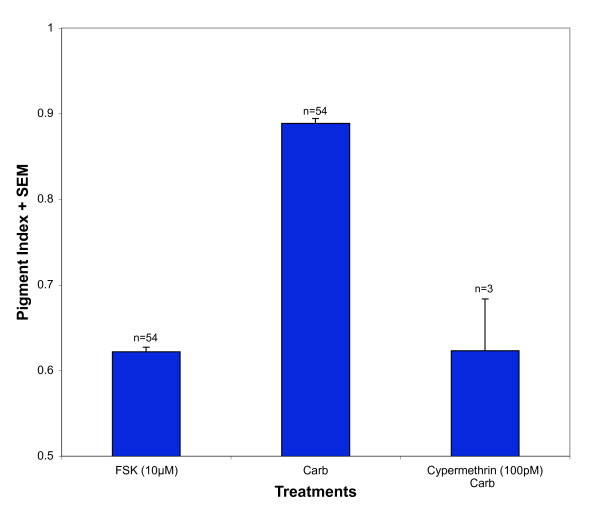
**Calcineurin-inhibitor cypermethrin blocks carbachol-induced dispersion**. Isolated RPE cells were first treated with 10 μM forskolin for 45' to induce aggregation. Following aggregation, carbachol (100 nM) was tested along with the calcineurin inhibitor cypermethrin (100 pM). Cypermethrin-treated cells experienced 100% inhibition in dispersion.

Another Ca^2+^-dependent effector tested was the protein phosphatase calcineurin. The calcineurin inhibitor cypermethrin (100 pM) completely inhibited carbachol-induced pigment granule dispersion (Figure 5). The cypermethrin condition (0.62 ± 0.06, n = 3) was not significantly different from the FSK condition (0.62 ± 0.01, n = 54), but was different from the carbachol condition (0.89 ± 0.01, n = 54).

## Discussion

The results indicate that an elevation of intracellular Ca^2+ ^is required for carbachol-induced pigment granule dispersion. Furthermore, the necessary Ca^2+ ^may be derived entirely from intracellular stores, and one effector activated by Ca^2+ ^is the protein phosphatase calcineurin. In contrast, our results using inhibitors and activators of PKC suggest that the activity of this enzyme is neither necessary for carbachol-induced pigment granule dispersion nor sufficient to drive pigment granule dispersion.

Carbachol-mediated dispersion requires activation of the IP_3 _receptor [18], suggesting that intracellular Ca^2+ ^stores are accessed following muscarinic receptor activation [17]. Chelating intracellular Ca^2+ ^with BAPTA completely blocks carbachol-induced dispersion. That this Ca^2+ ^is most likely coming from intracellular stores (regulated by the IP_3 _receptor) and not from the extracellular environment is supported by the observations that carbachol-induced dispersion is blocked by the IP_3 _receptor antagonist 2-aminoethoxydiphenyl borate [18] and eliminating extracellular Ca^2+ ^or blocking its entry across the plasma membrane failed to inhibit dispersion. There are many Ca^2+^-requiring mediators that could be involved in carbachol-induced dispersion, including the Ca^2+^-calmodulin-dependent protein phosphatase calcineurin. Since cypermethrin inhibits calcineurin activity, finding that cypermethrin blocks carbachol-induced pigment granule dispersion suggests that calcineurin is involved. This result correlates with work done by Thaler and Haimo [21]. Studying melanophores, they found a role for calcineurin in pigment aggregation. Furthermore, García [22] found that okadaic acid induces aggregation in RPE cells of green sunfish, suggesting a role for protein phosphatases in maintaining pigment granule dispersion.

One possible role for calcineurin is to remove phosphate groups added to proteins by PKA [23]. When FSK is applied to cells, cAMP levels increase [12], leading to PKA activation [22] and eventual aggregation of pigment granules. In the RPE pigment motility system, the substrate phosphorylated by PKA is as yet unknown. Prior to dispersion, cAMP levels drop and likewise PKA activity. Increased Ca^2+ ^concentrations inside the cell may lead to calcineurin activation that may remove phosphate groups from substrates of PKA, enabling pigment granule dispersion to occur. Our finding that cypermethrin inhibits carbachol-induced dispersion is consistent with this model.

As stated before, inhibition of PLC blocks carbachol-induced dispersion [18]. Since PLC cleaves PIP_2 _into IP_3 _(leading to Ca^2+ ^mobilization) and DAG, and DAG and Ca^2+ ^together activate protein kinase C, protein kinase C seemed likely to be a mediator of carbachol-induced dispersion [24]. This hypothesis seemed the more likely since in some species, pigment dispersion in melanophores appears to be regulated by PKC [25,26]. However, our results from three separate experiments failed to support a role for PKC in carbachol-induced dispersion. Treatment with the PKC activator PMA did not induce pigment granule dispersion. Neither of the PKC inhibitors tested (bisindolylmaleimide and staurosporine) inhibited carbachol-induced pigment granule dispersion at most concentrations tested. Cells treated with bisindolylmaleimide II at 200 nM experienced a 20% inhibition in dispersion relative to the carbachol-treated cells when data from the latter were compiled from all experiments. However, when comparison was made only to the carbachol-treated cells from the experiment in which the 200 nM concentration was tested, the difference in mean pigment indices was not found to be statistically significant. This case was the only one in which the compilation of the data yielded a statistical outcome different from analysis of the data within single experiments. Nevertheless, it remains possible that PKC has a role in carbachol-mediated dispersion which parallels the main pathway linking receptor activation to pigment granule movement. It should be mentioned that definitive evidence that PKC is expressed in bluegill RPE has yet to be found. We have found that PKC-α and PKC-β were expressed in bluegill brain, but not in bluegill RPE (unpublished results). These findings are consistent with work reported by others. In the retina of zebrafish, PKC labelling is observed in the neural retina, but not in the retinal pigment epithelium [27].

**Figure 6:**
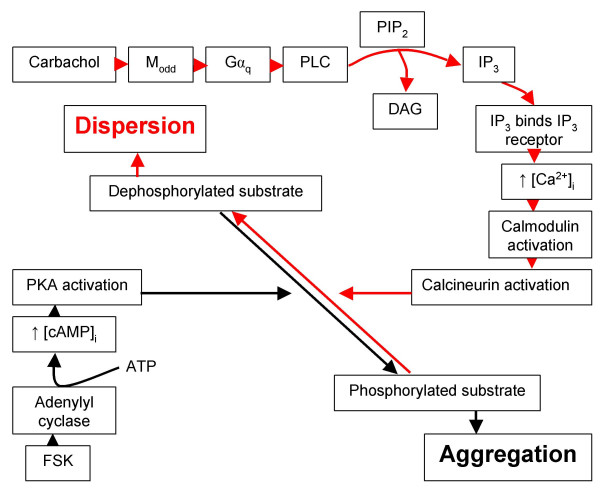
**Possible signaling pathway for carbachol-induced pigment granule dispersion in bluegill RPE**. In conjunction with previous evidence, a pathway that follows carbachol and forskolin (FSK) treatment is proposed. Arrows in red represent the pathway involved in dispersion; whereas, black arrows represent the aggregation pathway. All of the mediators shown have been tested with the exception of calmodulin, which may be required for calcineurin activation [28]. The specific phosphorylated/dephosphorylated substrate(s) is(are) unknown. Abbreviations used are as follows (listed alphabetically): **ATP**, adenosine triphosphate; **Ca**^**2+**^_**i**_, intracellular free calcium ions; **cAMP**_**i**_, intracellular cyclic adenosine monophosphate; **DAG**, diacylglycerol; **FSK**, forskolin; **Gα**_**q**_, alpha subunit of the G_q _family of GTP-binding proteins; **IP**_**3**_, inositol trisphosphate; **M**_**odd**_, muscarinic acetylcholine receptor subtype 1, 3, or 5; **PIP**_**2**_, phosphatidyl inositol bisphosphate;**PKA**, cAMP-dependent protein kinase;**PLC**, phospholipase C.

Although much remains to be discovered, our results have increased our understanding of the pathway from carbachol to pigment granule dispersion. We propose the following model: First, carbachol binds an M_odd _muscarinic receptor leading to PLC activation [17,18]. PLC then cleaves PIP_2 _into DAG and IP_3_. A role for DAG in pigment movement in RPE has not been revealed. In contrast, IP_3 _binds its receptor, thus releasing Ca^2+ ^from intracellular stores [18]. Although the conclusion requires support from Ca^2+ ^imaging, our data indicates that Ca^2+ ^released from such stores is sufficient for downstream mediator activation; in other words, extracellular Ca^2+ ^is not required. Increasing cytosolic Ca^2+ ^leads to calcineurin activation. Calcineurin removes phosphate groups added by PKA on an as yet unidentified protein which functions as a molecular switch regulating the pigment granule position in the retinal pigment epithelium. Refer to Figure 6 for the proposed carbachol-induced dispersion pathway.

## Conclusion

Our results indicated that in the RPE of bluegill, carbachol-induced pigment granule dispersion is a process that requires intracellular, but not extracellular, Ca^2+^. Our evidence also supports a pigment dispersion model that requires calcineurin but not protein kinase C.

## Methods

### Experimental animals

The experimental animals were maintained as described previously [17]. All experiments were performed following protocols approved by the Institutional Animal Care and Use Committee (IACUC) of Texas State University- San Marcos (Protocol # 069744F82). Bluegill (*Lepomis macrochirus*) were purchased from Johnson Lake Management, San Marcos, TX. All fish were acclimated at least two weeks prior to experimentation in indoor, aerated aquaria on a 12 hour: 12 hour (light/dark) cycle.

To extract RPE, fish were captured 6 hours into the light cycle and dark adapted in a room void of light. In the dark room, the fish were allotted thirty minutes to adjust to the darkness in aerated aquaria. After thirty minutes, the spinal cord was severed, and the fish were double-pithed under dim, incandescent light (≤ 2 lux). Light was measured using a Lutron LX-101 lux meter (Lutron, Coopersburg, PA). The eyes were then removed and hemisected along the equatorial axis. The anterior portion of the eye was discarded. The retina was removed from the eyecup, and the RPE was flushed out using bicarbonate-buffered Ringer prepared the day of the experiment [17]. All chemicals were purchased from Sigma-Aldrich, St. Louis, MO. The Ringer's solution contained 24 mM NaHCO_3_, 3 mM HEPES (free acid), 116 mM NaCl, 5 mM KCl, 1 mM NaH_2_PO_4_·H_2_O, 26 mM dextrose, 1 mM ascorbic acid, 1.12 mM MgSO_4_, 1 mM EGTA, and 1.8 mM CaCl_2_, titrated to pH of 7.4 using 1 M NaOH. The buffer was gassed with 95% air/5% CO_2 _for at least 15' prior to the dissection and throughout the experiment to maintain a pH of 7.2. For one experiment, Ca^2+^-free Ringer was prepared as above with the omission of CaCl_2_.

Once the RPE had been isolated, excess Ringer was removed, and forskolin (10 μM) was applied to induce aggregation. The cells were then incubated for 45' in a humidified chamber gassed with a mixture of 95% air and 5% CO_2_on a gyratory shaker (50 rpm) as in González *et al*. [17].

After incubation in forskolin, approximately one third of the collected RPE was fixed using a 2× stock solution of fixative prepared in phosphate buffered saline (PBS). PBS was prepared as 137 mM NaCl, 2.7 mM KCl, 4.3 mM NaH_2_PO_4_·H_2_O, and 1.4 mM KH_2_PO_4 _in purified water (resistivity 18 Mohms). Purified water was obtained from a NANOpure Infinity Laboratory Water System. After dilution with an equal volume of Ringer's solution containing tissue, the final concentration of fixative was 0.5% glutaraldehyde, 0.5% paraformaldehyde, and 0.8% potassium ferricyanide.

The remaining cells were divided between two weigh boats and washed clean of forskolin using Ringer. Ringer's solution was pipetted into and out of the weigh boats; about 3 volumes were exchanged. Tissue pieces for both treatment groups were then placed in microcentrifuge tubes containing either 50 μl of 1 μM carbachol and 450 μl of Ringer's solution or 50 μl of 1 μM carbachol, 400 μl of Ringer's solution, and 50 μl of 10× experimental drug (see **Pharmacological Agents **below). Following an additional 45' incubation, the remaining samples were fixed as described above.

### Pharmacological Agents

To study the requirement for extracellular and intracellular Ca^2+^, verapamil and BAPTA-AM (both of which were prepared fresh the day of experimentation) were used, respectively. Cypermethrin (from 1 nM stock solution) was used to inhibit calcineurin (PP2B). Staurosporine, bisindolylmaleimide II, and phorbol esters were used to investigate a role for PKC activity. Staurosporine (Sigma) was divided into 10 μM aliquots in DMSO. Bisindolylmaleimide II (Sigma) was dissolved in DMSO to make a 1 mM stock solution, which was then divided into aliquots and frozen. Phorbol 12-myristate 13-acetate (Sigma) was prepared as a 100 μM stock and divided into aliquots. All inhibitors prepared in DMSO were stored at -20°C, then diluted in Ringer's solution just before use. A complete list of agents used can be found in Table 1.

**Table 1:** Pharmacological agents used to uncover ions and molecular mediators involved in carbachol-induced pigment granule dispersion

**Drug**	**Target/effect**	**Concentration**	**Reference**
Verapamil	L-type Ca^2+ ^channel antagonist	10 μM, 100 μM	[29]
BAPTA-AM	Intracellular Ca^2+ ^chelator	1 μM, 30 μM	[21]
Cypermethrin	PP2B (calcineurin) inhibitor	100 pM	[30]
Staurosporine	PKC inhibitor	100 nM, 0.5 μM, 1 μM	[25]
Bisindolylmaleimide II	PKC inhibitor	20 nM, 200 nM, 2 μM	[31]
Phorbol 12-myristate 13-acetate	PKC activator	1 μM, 10 μM, 100 μM	[25]

Drug concentrations used are justified by the articles referenced. All agents were purchased from Sigma-Aldrich (St. Louis, MO).

### Microscopy and statistical analysis

Fixed RPE was placed onto a slide and chopped into smaller fragments using a coverslip. For each treatment sample, thirty cells were measured from each fish, and a minimum of three fish was used in each experiment. Measurements were made using a Zeiss phase-contrast light microscope equipped with an ocular micrometer. Cells were measured if they appeared to be whole, that is, if they had the phase-bright base typical of an intact cell and long apical processes giving an overall length of at least 50 μm. Cells were only measured if they had at least three apical processes.

A pigment index (PI) was calculated by dividing the distance from the base of the cell to the farthest dispersed pigment granule by the total length of the cell. Analysis of variance (ANOVA) was performed among the mean pigment indices of each treatment group. Differences were considered significant when p < 0.05. Tukey *post hoc *analysis was performed to determine which treatments differed. Prior to analysis, the two control conditions (FSK and carbachol-alone) were combined from all experiments. When experimental conditions were found statistically significantly different from the carbachol control condition, a percent inhibition was calculated. To calculate percent inhibition, first the difference between experimental treatment and FSK treatment was found. This value was then divided by the difference between FSK and carbachol conditions and multiplied by 100. Finally, the resulting value was subtracted from 100 to yield percent inhibition.

## Authors' contributions

ASJ conducted all of the pharmacological experiments, the statistical analysis and prepared the first draft of the manuscript. DMG conceived of and supervised the study, secured funding, and prepared the manuscript for publication.
